# A novel empirical wavelet SODP and spectral entropy based index for assessing the depth of anaesthesia

**DOI:** 10.1007/s13755-022-00178-8

**Published:** 2022-06-06

**Authors:** Thomas Schmierer, Tianning Li, Yan Li

**Affiliations:** School of Mathematics, Physics and Computing, University of Southern Queensland, Darling Heights, Australia

**Keywords:** Depth of anaesthesia, Statistical model, Empirical wavelet transform, Second order difference plot

## Abstract

The requirement for anaesthesia during modern surgical procedures is unquestionable to ensure a safe experience for patients with successful recovery. Assessment of the depth of anaesthesia (DoA) is an important and ongoing field of research to ensure patient stability during and post-surgery. This research addresses the limitations of current DoA indexes by developing a new index based on electroencephalography (EEG) signal analysis. Empirical wavelet transformation (EWT) methods are employed to extract wavelet coefficients before statistical analysis. The features Spectral Entropy and Second Order Difference Plot are extracted from the wavelet coefficients. These features are used to train a new index, SSE_DoA_, utilising a Support Vector Machine (SVM) with a linear kernel function. The new index accurately assesses the DoA to illustrate the transition between different anaesthetic stages. Testing was undertaken with nine patients and an additional four patients with low signal quality. Across the nine patients we tested, an average correlation of 0.834 was observed with the Bispectral (BIS) index. The analysis of the DoA stage transition exhibited a Choen's Kappa of 0.809, indicative of a high agreement.

## Introduction

Anaesthesia is a crucial component of modern surgical procedures. Bowdle [1] observes that accurate monitoring of anaesthetic levels during surgery is associated with improved patient outcomes, reduced likelihood of operative awareness, and faster recovery following surgery. Traditional measures of determining DoA include observation of physical traits such as muscle reflex actions, pulse, and blood pressure. Despite this, Diykh et al. [2] suggested that the variation in individual patient response to anaesthetic agents prevents reliable and uniform observations of the DoA based on these measures. Alternatively, an accurate assessment of the DoA is achievable by examining patterns in electroencephalography (EEG) [3, 4]. EEG signals can reflect the intrinsic connections and functions of the brain [5, 6]. The observed regularity and patterns of EEG signals can provide insights into the level of unconsciousness. Furthermore, John [7] and Saadeh, Khan and Altaf [8] observed that EEG-based DoA monitoring methods' reliability is independent of the anaesthetic agent used.

A range of EEG-based DoA monitoring algorithms are currently in operation throughout modern surgical procedures. The bispectral index (BIS) is the current market-leading application for measuring DoA and is considered a suitable comparative index in model building for DoA applications [9, 10]. The BIS index processes the EEG signal based on time and frequency components and returns a value between 0 and 100 on a BIS monitor. This index reflects the patient’s anaesthetic states during surgery and may assist the anaesthetist in providing appropriate medication for maintaining appropriate levels of unconsciousness. In recent studies, Li and Wen [11], Diykh et al. [2], and Nguyen-Ky et al. [12, 13] addressed the limitations of its observed susceptibility to interference from noise, time delay, and inconsistency between patients in the BIS index. Continual research and development in refining EEG based DoA algorithms are necessary to assist medical professionals in the care of patients undergoing surgery.

Recent work by Liu et al. [14] in applying empirical wavelet transform (EWT) to decompose seismic data has shown promising results. We were motivated by the effectiveness of EWT for signal decomposition in our application with EEG signal for DoA estimation. In this paper, a new index for estimating DoA based on EEG signals is proposed to utilise EWT and statistical features and use a window technique to divide the EEG signal into segments. Each EEG segment is then partitioned into ${\varvec{n}}$ EWT subbands to produce a vector of wavelet coefficients for each subband. Two statistical features, Second Order Differential Plot (SODP) and Spectral Entropy (SE), are extracted from the highest order subband of each segment. A new function of a DoA estimate (SSE_DoA_) is designed with a Support Vector Machine (SVM) utilising a linear kernel function. The new index is evaluated using a recorded EEG signal and the BIS index from 13 subjects. Pearson correlation, r, and Mean Squared Error (MSE) are used to evaluate the agreement between the BIS and the new index, SSE_DoA_. Supervised machine learning utilising 80% confidence interval rule classification based on the SSE_DoA_ index is implemented to evaluate the DoA states classification of the transition. Cohen's Kappa coefficient is used to determine the level of agreement between the predicted and actual DoA state classification for the test patients. The actual DoA state is determined based on the anaesthetist's notes and examination of the BIS of the patient at that time. This classification method is a novel application based on the work by Nguyen-Ky et al. [15].

**Fig. 1:**
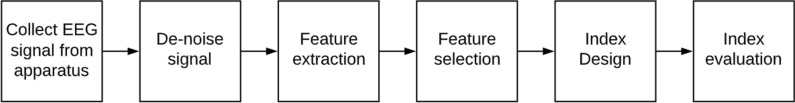
Structure Diagram for EEG based DoA assessment methods

A variety of DoA methods based on EEG recordings have been developed. The purpose of ongoing research in this area is to refine an accurate index to monitor the DoA. The general structure of DoA assessment models follows the flow chart in Fig. 1. The core feature extraction and index design processes contribute to the significant difference between DoA assessment methods.

Saadeh et al. [8] proposed a method for DoA classification based on spectral estimation methods, including spectral edge frequency, beta ratio, and spectral energy; these selected features are known to be present in the BIS algorithm [8, 11, 16, 17] and have been shown to develop indices with close representation to the BIS index. This work implemented a band-pass filter for signal denoising, and a fine decision tree classifier was used in the DoA index design. In classifying the DoA states into four levels of unconsciousness, an accuracy of 92.2% was observed in that study. The outcome added strength to the validity of the selected feature extraction methods and their associated similarity with the BIS index. The spectral density methods utilised the extracted information from a signal as a stochastic process that can describe the distribution of the signal's power in its frequency domain. The randomness of EEG signals limits this characteristic to an estimation based on the sequence of time samples. The spectral density process assumes that the signal can be described parametrically from a linear system driven by white noise [17].

Different levels of cognitive awareness are known to be associated with varying patterns of EEG signal complexity. Consequently, entropy-based feature extraction methods are specifically suited to decoding an EEG signal through the detection of its complexity [18–20]. More recently, Almeer [20] implemented multi-scale sample entropy to extract complexity information from several EEG channels simultaneously for DoA analysis. In this work, index design was implemented with an artificial neural network. Classification of the DoA states observed an overall accuracy of 95% in that work. The computational intensity of the algorithm design was considered a limitation of this method.

Wavelet-based methods have been extensively utilised in EEG signal analysis for various biomedical applications. The computational efficiency and capacity to discriminate both the temporal and spectral domain features of signals add value to this method [21, 22]. Furthermore, wavelet-based approaches are not inhibited by time–frequency compromises inherent to other methods [17]. Due to the extent of features extracted through these methods, feature selection plays an increasingly significant role in research methodologies. The application of EWT to EEG signals for DoA analysis is considered novel. The success of this method in other fields of EEG signal analysis was a motivation for this research.

Li and Wen [11] implemented linear regression following the extraction of mobility, permutation entropy and Lempel–Ziv complexity in DoA estimation with an EEG signal. Their proposed method is considered the benchmark in index design due to the observed effectiveness because of the modelling technique's simplicity and computational efficiency. This index observed close representation to the BIS index with an average Pearson correlation across 19 patients of 0.809. Furthermore, an earlier time response of 25 to 264 s was observed in that study.

Alternatively, Support Vector Machines (SVM) is a popular model building technique for EEG analysis in both regression and classification systems [8, 23]. Recently, Tapani, Vanhatalo and Stevenson [24] applied an SVM index design to time and frequency domain features in seizure classification for neonatal subjects. The method outperformed human expert classifications in 73/79 cases.

## Methods

**Fig. 2:**
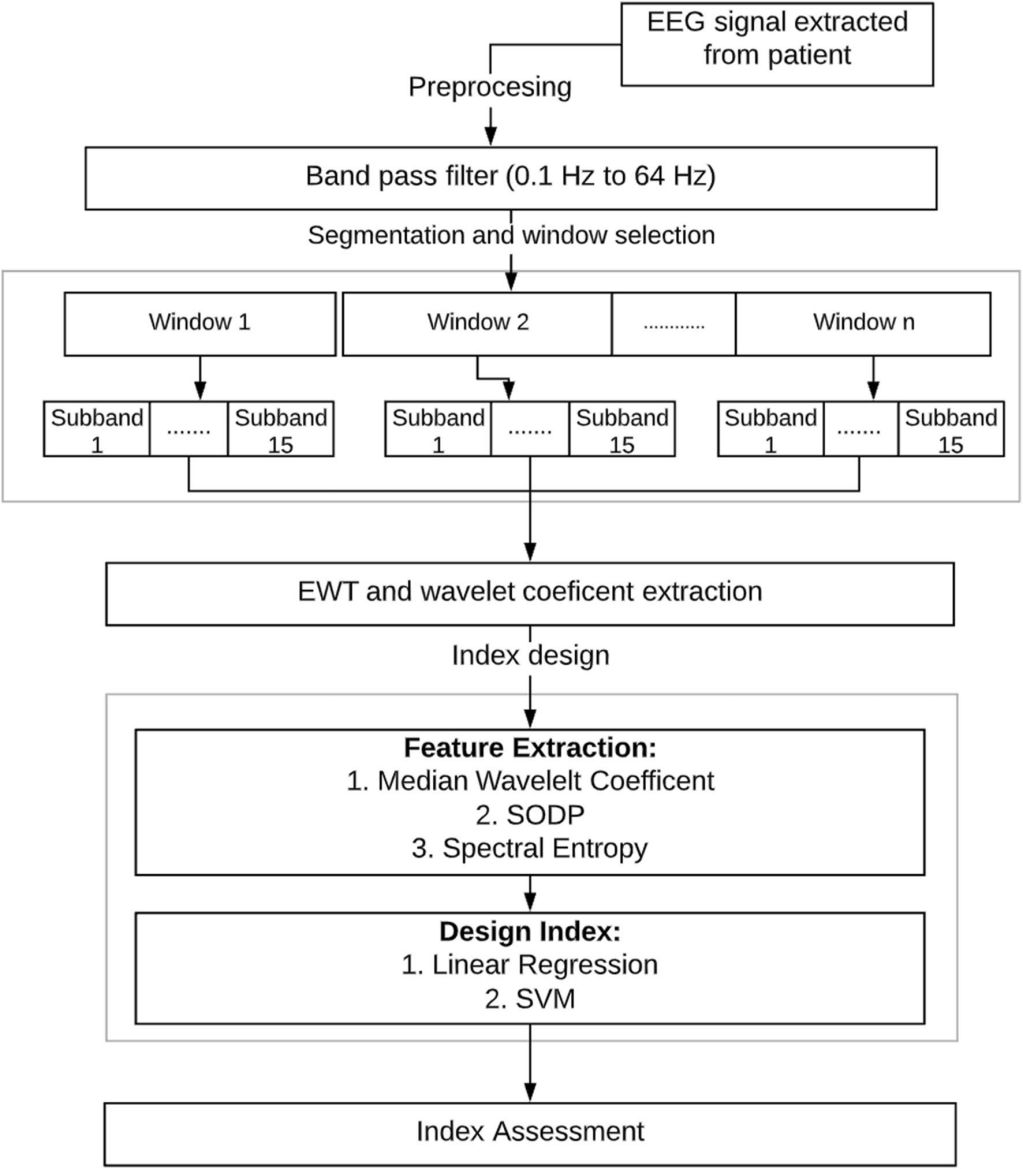
Block diagram of the proposed method to estimate the DoA

The proposed method for the new DoA index, SSE_DoA_, is based on statistical feature extraction that is derived from the coefficients following EWT, as illustrated in Fig. 2. The application of EWT processing employed here is the first paper that uses this process in DoA assessment. The EEG signal is initially partitioned into small segments utilising the window segmentation method discussed by Diykh, Li, Wen and Li [2] and Li [25]. The authors observed that a 56 s EEG signal window with a 55-s overlap produced optimal results and was hence applied in this paper. The incoming signal is decomposed using EWT into ${\varvec{n}}$ subbands, where the wavelet coefficients are extracted for analysis. The SE and SODP parameters are calculated from these windows and averaged over each segment. Index design is undertaken with an SVM utilising a linear kernel function. The novel work in this research is the application of EWT for preliminary EEG signal decomposition for the DoA analysis.

### Feature extraction and feature selection

The spectral entropy (SE) of a signal is the measure of its spectral power distribution. The concept is based on information entropy, a subset of information theory. The SE treats the signal's normalised power distribution in the frequency domain as a probability distribution and calculates the spectral entropy of the signal. This property is seen to be useful for feature extraction in biomedical applications [26].

The equations for the power spectrum and probability distribution for a signal underpin the spectral entropy function. For a given signal *x(n)*, *X(n)* is the discrete Fourier transform of *x(n), n* = *1, 2, …, N*.

P(n), the probability distribution, is thus:

1 $$P\left(m\right)=\frac{X(n)}{{\sum }_{i}X(i)}$$

where $$\sum_{m=1}^{M}P(n)=1$$

Hence, the spectral entropy, SE, at time t, can be expressed as:

2 $${SE}_{n}(t)= -\sum_{n=1}^{N}P\left(t,n\right){\mathrm{log}}_{2}P(t,n)$$

The SODP (Second Order Difference Plot) method has been shown to be effective in the analysis of the EEG signal for the DoA [25]. The SODP of intrinsic mode functions provides the elliptical structure, and the feature space is formed using ellipse area parameters. The moving vector of the EEG signal encompassing the 56-s window size is used as the input in this study.

SODP is calculated discreetly for each second in the input window [27]. The SODP for the signal, *x(n),* is found by plotting $X\left(n\right)=x\left(n+1\right)-x(n)$ against $Y\left(n\right)=x\left(n+2\right)-x(n+1)$, where:

3 $$SODP=|\mathrm{log}(3\pi \sqrt{\left(S{X}^{2}+S{Y}^{2}+D\right)\left(S{X}^{2}+S{Y}^{2}-D\right)}|$$

And $SX$ and $SY$ are defined as; $SX=\sqrt{\sum_{N=0}^{n-1}\frac{X{\left(n\right)}^{2}}{N}}$ and $SY=\sqrt{\sum_{N=0}^{n-1}\frac{Y{\left(n\right)}^{2}}{N}}$

$SXY$ is defined as:

4 $$SXY=\frac{1}{N}\sum (X\left(n\right)*Y\left(N\right))$$

The distance, D, is hence calculated as;

5 $$D=\sqrt{\left(S{X}^{2}+S{Y}^{2}\right)-4(S{X}^{2}S{Y}^{2}-SX{Y}^{2})}$$

The mean values of the SE and SODP over the 56-s window were used as the extracted features.

In the initial stages of feature extraction, EWT was applied to the Channel 2 signal in order to separate this signal into separate subbands. The previous feature extraction methods were then applied to select meaningful features. The implementation of EWT in this work is considered novel in terms of measuring the DoA. However, it has been successfully implemented in other EEG applications. The extraction of a subband allows for the analysis to be performed on the coefficients of a single subband in isolation. In this way, the refined subband signal can exhibit the true nature of the brain function and can illustrate the DoA situation more accurately.

Existing research typically extracts frequency bands by implementing frequency band filters. This method requires a signal to be denoised prior to analysis, resulting in a substantial time delay. Implementing EWT methods does not require discreet denoising as the process itself can denoise the signal through the generation of subbands.

The EWT process was implemented in MATLAB using the algorithm by Gilles [28]. A fundamental characteristic of the EWT method is the automatic identification of the wavelet boundaries. This adaptive method constructs the wavelet filter bank on the basis of the information contained in the signal. The inability to specify these boundaries is a distinguishing difference between EWT and the conventional frequency band filter. The wavelet filters are constructed by applying Fourier boundary detection to each 56-s window of the EEG signal. Different portions of the spectrum corresponding to different modes are separated by identifying the Fourier supports [28].

The EWT, ${\mathcal{W}}_{f}^{\varepsilon }(n, t)$, is defined in the same manner as the classical wavelet transform. Here the coefficients are the inner products of the empirical wavelets [28]:

6 $${\mathcal{W}}_{f}^{\varepsilon }\left(n, t\right)=\langle f,{\psi }_{n}\rangle =\int f\left(\tau \right)\overline{{\psi }_{n}\left(\tau -t\right)}d\tau$$

The approximation coefficients, given by ${\mathcal{W}}_{f}^{\varepsilon }(0, t)$, is the inner product with the scaling function:

7 $${\mathcal{W}}_{f}^{\varepsilon }\left(0, t\right)=\langle f,{\phi }_{1}\rangle =\int f\left(\tau \right)\overline{{\phi }_{1}\left(\tau -t\right)}d\tau$$

where ${\widehat{\psi }}_{n}(\omega )$ and ${\widehat{\phi }}_{1}(\omega )$ are defined according to the idea used in the construction of Littlewood-Paley and Meyer's wavelets [29]. Therefore:

8 $$f\left(t\right) =\widehat{{\mathcal{W}}_{f}^{\varepsilon }}\left(0, \omega \right)*{\widehat{\phi }}_{1}\left(\omega \right)+\sum_{n-1}^{N}\widehat{{\mathcal{W}}_{f}^{\varepsilon }}\left(n, \omega \right)*{\psi }_{n}\left(t\right)$$

Therefore, the empirical mode ${f}_{k}$, as defined by Gilles [28], is:

9 $${f}_{0}\left(t\right)= {\mathcal{W}}_{f}^{\varepsilon }\left(0, t\right)*{\phi }_{1}\left(t\right)$$

10 $${f}_{k}\left(t\right)= {\mathcal{W}}_{f}^{\varepsilon }\left(k, t\right)*{\psi }_{k}(t)$$

In implementing this function, a vector of the non-denoised signal, representative of the 56-s window, was used as the EWT input. A range of window sizes was investigated based on the recommendation of appropriate literature [25].

The R^2^ value shows the degree of variance between the extracted feature and the BIS value. The extracted features are calculated over the 15 EWT subbands. Analysis of the correlation between the extracted features and the BIS and their effectiveness for analysis. R2 is defined as:

11 $${R}^{2}=1-\frac{{\sum }_{i}{\left({y}_{i}-{f}_{i}\right)}^{2}}{{\sum }_{i}{\left({y}_{i}-\overline{y }\right)}^{2}}$$

where ${y}_{i}$ is the known BIS value, ${f}_{i}$ is the new index, and $\overline{y }$ is the mean of ${y}_{i}$. The coefficient of determination ranges between 0 (no relationship) and 1 (a perfect relationship). In this case, the higher the observed R squared value for a given feature, the more useful this feature (or parameter) set is expected to be in modelling the state of unconsciousness.

### Regression models

Machine learning algorithms have been extensively used in signal classification for EEG analysis. Two different machine learning algorithms were applied: linear model regression (LM) and support vector machine (SVM) are applied for training using the training datasets, and the model is evaluated using the testing datasets. Combining the features developed utilising the EWT wavelet subbands can describe the difference in the aesthetic states.

Linear regression models are those that can be described by the generalised equation below:

12 $${y}_{i}={b}_{0}+{b}_{1}{x}_{i1}+{b}_{2}{x}_{i2}+\dots +{b}_{P}{x}_{iP}+{e}_{i}$$

where ${y}_{i}$ represents the response for the ${i}^{th}$ sample based on the input features ${x}_{i1}$ to ${x}_{iP}$. The coefficients of the predictors are represented by ${b}_{1}$ to ${b}_{P}$ and ${b}_{0}$ represents the model’s constant. ${e}_{i}$ represents the error term. These models attempt to estimate the parameters ${b}_{n,}$ such that the mean square error (MSE) for the training data is at a minimum.

SVM models utilise the squared residuals when they are comparatively small and adapt the function to employ the absolute residuals when larger. Data points outside the user-defined threshold contribute a linear scale amount, while those within the threshold do not contribute to the regression fit. Consequently, large outliers have a limited effect on modelling. Furthermore, data points that the model fits overly well do not contribute to the model. The SVM employs the loss function, ${\varvec{\epsilon}}$, and applies a penalty factor. Thus, the SVM regression model attempts to minimise the function:

13 $$Cost\sum_{i=1}^{n}{L}_{\epsilon }({y}_{i}-\widehat{{y}_{i}})+ \sum_{j=1}^{P}{\beta }_{j}^{2}$$

where $Cost$ parameter is the user-defined penalty to larger residuals. The predictor based on the SVM can be defined as:

14 $$f\left(u\right)= {\beta }_{0}+\sum_{i=1}^{n}{\alpha }_{i}K({x}_{i},u)$$

where $f(u)$ is the predictor and $u$ is the vector of explanatory variables. The function $K\left({x}_{i}, u\right)$ defines the kernel function. In this research, a linear kernel function was employed.

A moving average was applied to improve the stability of the developed index for both the SVM and linear models. The moving average was applied over a 6-s window with a 5-s overlap, such that:

15 $$Inde{x}_{smoothed}\left(t\right)= \frac{{\sum }_{0}^{5}Index\left(t-i\right)}{6}$$

### Evaluation methods

The strength of the linear relationship between the BIS and the new index can be measured with Pearson correlation, *r*, and is defined as:

16 $$r= \frac{{\sum }_{N}\left(\left(x-\overline{x }\right)\left(y-\overline{y }\right)\right)}{\sqrt{{\sum }_{N}{\left(x-\overline{x }\right)}^{2}{\sum }_{N}{\left(y-\overline{y }\right)}^{2}}}$$

where $x$ is the DoA prediction for the new index and $y$ represents the corresponding BIS value for that time component. The means of $x$ and $y$ are represented by $\overline{x }$ and $\overline{y }$, respectively. The correlation coefficient represents the degree of the linearity between the predicted index and the BIS value, where a correlation of 1 represents a perfect linear relationship, and $-1$ represents a perfect inverse relationship. A correlation of 0 is indicative of no meaningful relation.

A complimentary evaluation method, Mean Square Error (MSE), is considered. The MSE measures the average squared difference between the estimated values and the actual value. RMSE is the square root of MSE, where MSE is defined as:

17 $$MSE=\frac{1}{N}{\sum }_{n=1}^{N}{\left({y}_{n}- {\widehat{y}}_{n}\right)}^{2}$$

### DoA states and transition analysis

In addition to regression analysis, the overall effectiveness of the new index in predicting the DoA states was also evaluated. This was achieved by developing a rule-based assessment process to translate the new index into a categorical DoA state descriptor. This evaluation method is an adaptation based on the work by Nguyen-Ky, Tuan, Savkin, Do and Van [15]. Li et al. [30] observe that the DoA states, as indicated by the BIS, correspond to the following ranges: 0–40 corresponds to deep anaesthesia (DA), 40–60 moderate anaesthesia (MA), 60–80 light anaesthesia (LA), and 80–100 awake (AW). A moderate anaesthesia depth is noted as corresponding to a suitable level for surgery.

**Fig. 3:**
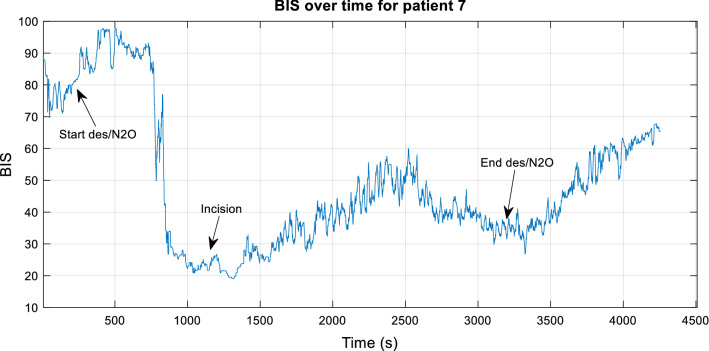
The BIS index over time for patient 7 with surgical notes indicated

The attending anaesthetist recorded surgery stages and drug administration relevant to the state of patient consciousness. The anaesthetic stage was assessed in conjunction with the BIS information and the recording physicians' surgical notes to determine the anaesthetic state of the patient during surgery, as shown in Fig. 3. This information was compiled for both the testing and training patient groups. For example, the rapid decrease in the BIS level at 500 s coincides with the observed stage transition into deep anaesthesia. The state of deep anaesthesia is observed to be relatively stable, extending into moderate anaesthesia from 1000 s to approximately 2000 s. This coincides with the recorded incision time and the ending of the anaesthetic agent at approximately 3000 s. The following period is considered the recovery period, and consciousness is to be restored. In this case, monitoring of the DoA ceases prior to the resumption of full cognitive awareness.

Rules are generated for the classification of anaesthetic states based on the training data distribution within each DoA state group. These rules are based on the new SVM index developed for the prediction of the DoA. A confidence interval of 80% based on the proportion of training information was implemented. This interval was considered appropriate as a minimal gap or overlap between states was observed. In addition, this rule produced intervals in very close agreement with the suggested BIS ranges for anaesthetic states.

Cohen's Kappa score (K) is computed to evaluate the degree of agreement between the predicted and actual DoA states generated by the new index [8]. K is defined as:

18 $$K= \frac{n{\sum }_{i=1}^{k}{n}_{ii-}{\sum }_{i=1}^{k}{n}_{.i}{n}_{i.} }{{n}^{2}-{\sum }_{i=1}^{k}{n}_{.i}{n}_{i.}}$$

where $i$ and $j$ represent the row and column indexes of the confusion matrix. Cohen’s Kappa score is used to justify the level of agreement between the observed state based on the anaesthetist notes and the rule-based assessment based on the new SSE_DoA_ index.

## Results

### Experimental data

The EEG signal data analysed in this study included 23 adult patients aged from 22 to 83 years and weighing between 60 and 130 kg. The EEG signal consisted of a two-channel signal recorded at a 128 Hz frequency. The BIS index values for patients are recorded during surgery at a rate of one value each second. The EEG signal was not denoised prior to analysis. Li and Wen [11] observed that Channel 2 EEG signal more closely represented the DoA and was used exclusively in this study. In addition, the anaesthetist’s notes with drug dosage and administration times were available and considered for 22 of the 23 patients.

The medical condition and procedure of data collection were explained to all patients. The consent, including all ethical issues, was obtained from all patients. The study was approved by the University of Southern Queensland Human Research Ethics Committee (No: H09REA029) and the Toowoomba and Darling Downs Health Service District Human Research Ethics Committee (No: TDDHSD HREC 2009/016).

#### Pre-processing

EEG signals are often disrupted or distorted by different types of noise, for example, from environmental or physiological sources. Environmental noise can be generated from powerline interference or other devices in the surgical theatre. Physiological noise can arise due to cardiac signals, movement, or poor device contact interference. A broad-spectrum band-pass filter is implemented on the original EEG signal to eliminate the impact of noise. The filter would select the EEG data samples between 0.1 and 64 Hz frequency ranges.

The 23 patients were randomly divided into two independent groups for training and testing. Feature extraction, feature selection, and index design were based on the selection of stable subsets of signal from the training patient group. Signal stability was considered based on the observed correlation between the anaesthetist’s notes and the recorded BIS value. Periods of rapid stage transition or poor signal quality were not used for model training. As defined by the BIS, approximately equal distribution across the full range of DoA stages was incorporated into the training data to reduce training bias. The results presented in this paper were based on the implementation of the new index on the testing patient group. Patients 1 to 10 were used to develop the training patient dataset, and patients 11 to 19 were used as the testing dataset for validation of the training model. The EEG data from patients 20 to 23 were observed to exhibit high levels of irregularity in the signal quality. They were used independently to test the effectiveness of the new index in the case of poor signal quality. Signal quality was measured during the surgery by the SQI (Signal Quality Index).

### Parameter selection

The Fourier boundaries were detected by computing the local maxima with the boundaries set as the smallest minima between consecutive maxima, known as "locmaxmin". This method was selected as it allows a finite number of subbands to be specified. The boundary detection was implemented based on the logarithm of the spectrum instead of the spectrum itself. Following the identification of the Fourier boundaries, the 1-dimensional (1D) wavelet coefficients for each subband were calculated.

The EWT method employed generated 15 subbands with feature extraction utilising only the highest order subband. EWT was applied to each 56-s window and extracted 15 subbands, whereby three features were extracted from each, generating 45 unique features in total per window. A range of EWT subbands were assessed for correlation with the features discussed above. In all cases, features extracted from the highest order EWT subband coefficients were seen to be most highly correlated with the BIS index. It was observed that when 15 EWT subbands were specified, the extracted features exhibited peak correlation with the BIS index; the effect was most significant for spectral entropy. The correlation of the BIS with both the SODP and median was seen to maintain uniformity independent of the number of extracted subbands. The selection of 15 EWT subbands was made regarding the effect of these respective features in the model building.

**Fig. 4:**
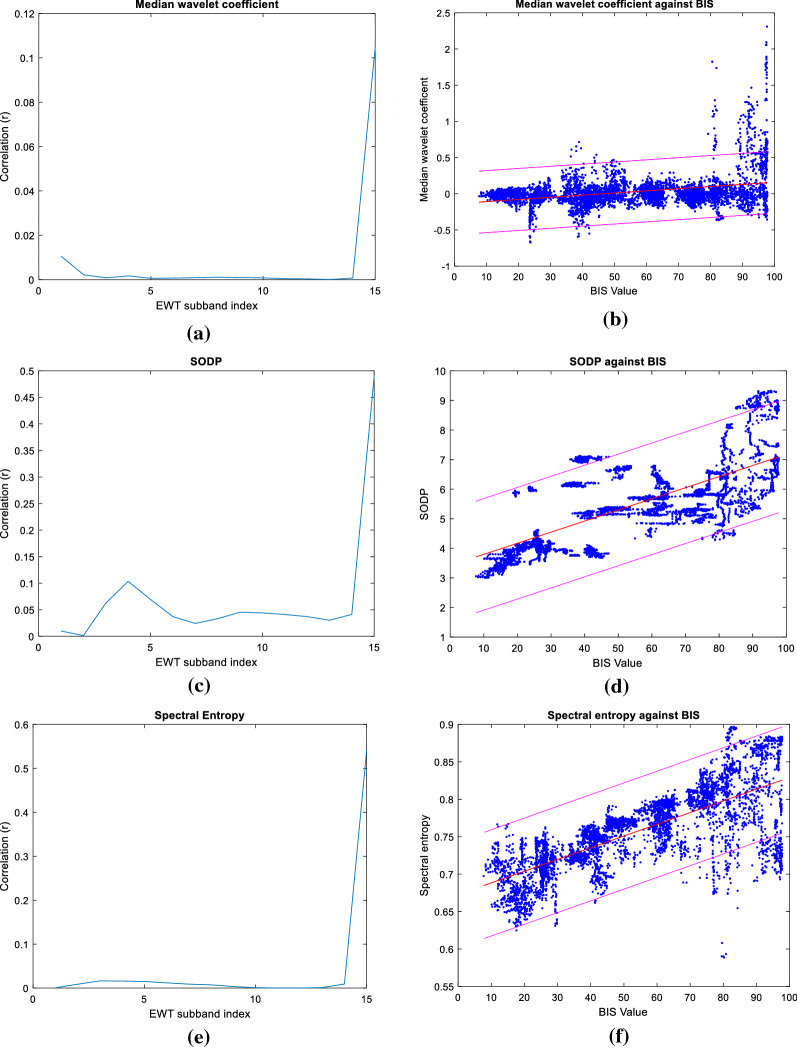
Comparison of EWT subbands: **a** Correlation of Median with the BIS feature for each EWT subband. **b** Scatter plot of the median EWT coefficient for the 15th band against the BIS index, 95% confidence interval shown. **c** Correlation of SODP with the BIS feature for each EWT subband. **d** Scatter plot of SODP for the 15th band against the BIS index, 95% confidence interval shown. **e** Correlation of Spectral Entropy with the BIS feature for each EWT subband. **f** Scatter plot of Spectral Entropy coefficient for the 15th band against the BIS index, 95% confidence interval shown

Figure 4a, c and e illustrate the R2 value for each respective EWT subband when 15 subbands are specified; the EWT subbands are presented in ascending order according to the respective detected Fourier boundaries with the highest frequency band assigned the highest reference index. The information presented is constructed using the training data set from 10 patients. Figure 4 illustrates the distribution of each feature extraction applied to the 15th EWT subband coefficient against the BIS value with the 95% confidence interval bands and the linear trend line indicated.

The Median value of the EWT coefficient is included in the model training as it has been observed to provide a stable baseline for the index design despite the low correlation with the BIS index. The peak correlation occurred with the 15th EWT band for all extracted features. The R squared value for Spectral Entropy and SODP with the BIS index is 0.52 and 0.47, respectively.

### Index design

Linear regression and SVM models are employed independently and evaluated to select the most suitable methods for the DoA index design. The outcomes are shown in Table 1 across three evaluation methods: Pearson correlation coefficient, MSE and RMSE. These models were developed using the three parameters, median wavelet coefficient, spectral entropy, and SODP. The SVM model is seen to be most effective when considering both the Pearson correlation, r, and the Mean Square Error (MSE). The highest correlation was observed at 0.943 for patient 15 with the SVM model. The smallest MSE of 79.12 was observed for patient 34 with the SVM model.

**Table 1:** Evaluation of testing patients

Model	Evaluation method	Patient index									
		11	12	13	14	15	16	17	18	19	Average
SVM	Pearson	0.866	0.808	0.786	0.859	0.943	0.925	0.768	0.787	0.848	0.834
LM	Pearson	0.798	0.769	0.773	0.793	0.940	0.799	0.639	0.612	0.600	0.753
SVM	MSE	111.5	88.2	239.0	364.8	131.1	177.6	188.6	79.1	116.2	172.5
LM	MSE	125.8	95.7	180.6	387.8	110.9	213.1	233.0	148.0	218.4	199.5
SVM	RMSE	10.56	9.39	15.46	19.10	11.45	13.33	13.73	8.90	10.78	12.75
LM	RMSE	11.22	9.78	13.44	19.69	10.53	14.60	15.26	12.17	14.78	13.84

### The agreement of the new DoA index using SVM with the BIS value

**Fig. 5:**
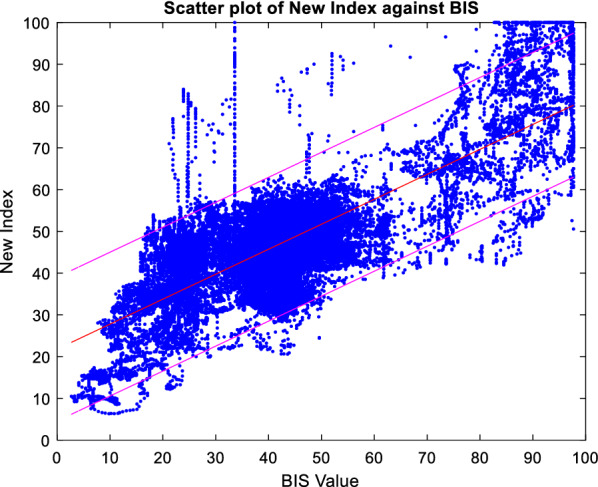
The scatter plot of the BIS index against the new index, SSE_DoA_, with 95% confidence interval, from testing results

**Fig. 6:**
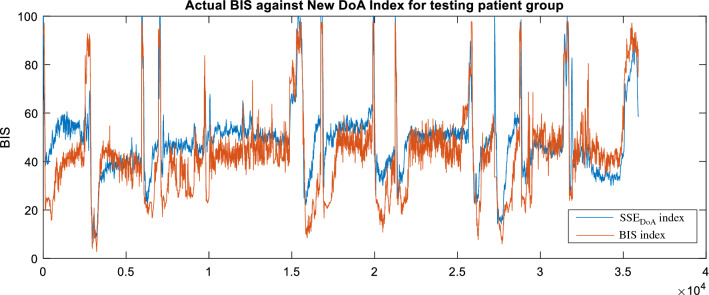
The BIS index against the new index, SSE_DoA_, from testing result for patients 11 to 23

The selected parameters of the median wavelet coefficient, spectral entropy and SODP are used to obtain the coefficients of a new DoA index using SVM, named SSE_DoA_. The new index is evaluated by comparing it with the BIS index using the testing EEG dataset. The average correlation of the SSE_DoA_ index for the testing results is 0.834, with a range from 0.768 (patient 17) to 0.943 (patient 15). The average RMSE is 12.74, with a range between 19.10 (patient 14) and 8.90 (patient 18). Figure 5 illustrates the SSE_DoA_ index against the BIS index for each observation in the training data set, including the poor-quality patient data. The SVM model is seen to produce a DoA index with a closer relationship with the BIS value. This is indicated by the proximity of the 95% confidence bounds to the trend line. The bulk of the patient data is in the BIS range of 20 to 60, corresponding to a moderate to deep anaesthetic state. In this region, the prediction is seen to be strong except for a few noticeable outliers that will be discussed in the following sections. The performance of the new DoA index for the entire testing patient group, inclusive of the low SQI EEG recordings, is shown in Fig. 6.

### Assessment of the DoA states and transition

In assessing the DoA states classification capacity of the new index, SSE_DoA_, association rules based on the anaesthetic states and EEG signal of stable anaesthesia periods were applied. Stability was considered based on the alignment of the anaesthetist notes and the BIS index. In addition, only patients’ EEG signal intervals that observed stable levels of SQI were considered.

**Fig. 7:**
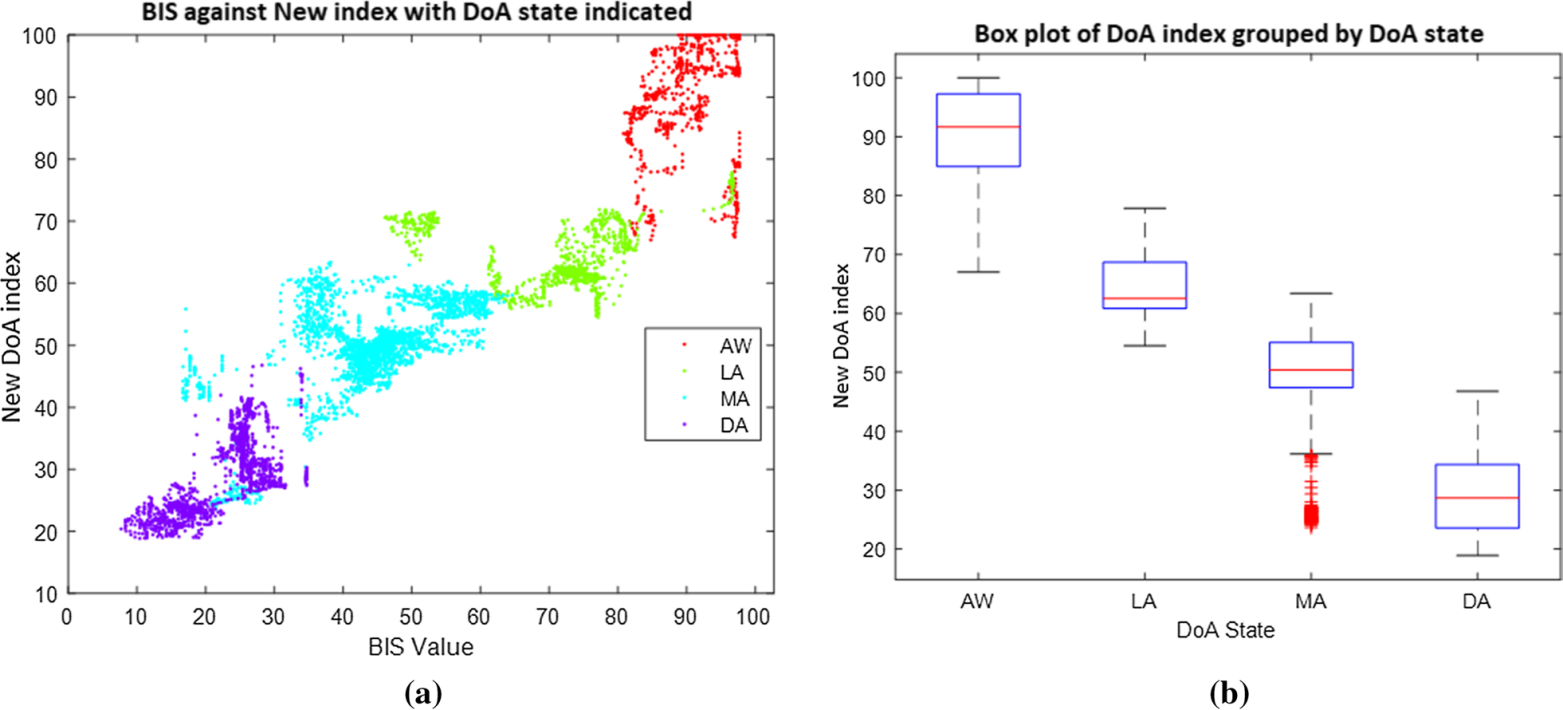
Comparison of DoA States: **a** BIS value against DoA Prediction with anaesthetic state indicated in colour. **b** Box plot illustrating the range of DoA prediction for each anaesthetic state for the training data from 10 patients/subjects

Rules for classifying the DoA states were generated by assessing the association between the DoA state and the SSE_DoA_ index from 5930 data points from the training EEG set. Rules implemented a boundary confidence interval of 80% of the proportion of training data for each state group. This interval provided minimal gap or overlap between states. Figure 7a shows the BIS value against the SSE_DoA_ index with the anaesthetic state indicated in colour for the training group. The box plot, Fig. 7b, illustrates the SSE_DoA_ index range for each anaesthetic state for the training dataset. The DoA state boundary rules are indicated in Table 2.

**Table 2:** DoA state rule boundaries based on the new SSE_DoA_ index

State	Lower bound	Upper bound
Awake (AW)	71.8993	100
Light anaesthesia (LA)	57.9933	70.4623
Moderate anaesthesia (MA)	43.2464	57.9141
Deep anaesthesia (DA)	21.446	38.6928

**Fig. 8:**
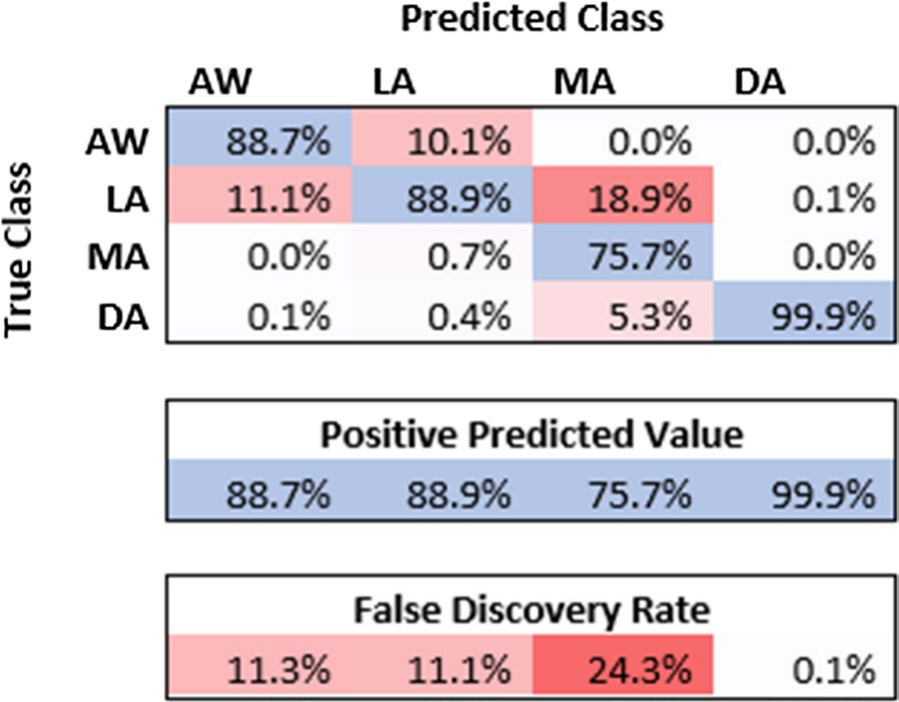
Confusion matrix for the prediction of DoA state based on the new index SSE_DoA_

In assessing the strength of these association rules, a selection of 4491 s of testing EEG data with stable DoA intervals was used. The association rules were applied directly to the SSE_DoA_ index for the testing data. Comparison between the actual DoA state and the predicted state indicates the very strong predictive power of the new index in assessing the DoA state. The classification accuracy of the DoA state is illustrated in the confusion matrix in Fig. 8. The Cohen's Kappa coefficient is ascertained to justify the effectiveness of the proposed index in determining the overall level of anaesthesia. The Cohen Kappa score of 0.809 is observed and considered a high-level agreement (between 0.8 and 1) [31, 32]

### Effectiveness of new index in the case of poor signal quality

As measured by the Signal Quality Index (SQI), signal quality was available for a limited subset of patients in the testing set. In several cases, information regarding the SQI may be used to suggest the reason behind the divergence between the predicted level of unconsciousness and the actual BIS level. Patients 20, 21, 22, and 23 were considered to be suitable examples of poor signal quality.

**Fig. 9:**
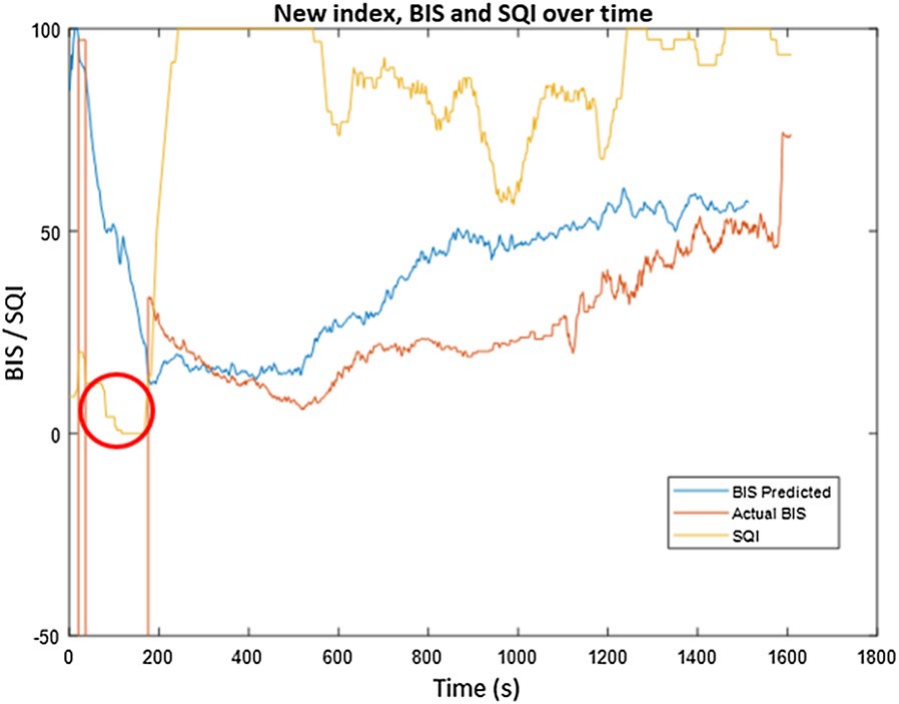
The SSE_DoA_ index is compared to the BIS value for patient 23 with SQI indicated

In the case of patient 23, a correlation of 0.760 is observed between the SSE_DoA_ index and the BIS index and is directly associated with the observed instances of poor signal quality. The BIS index, alongside the new SSE_DoA_ index and the SQI, is shown below in Fig. 9 for the duration of patient 23's surgery. The atypical information corresponding to the first 170 s when the BIS was observed outside acceptable bounds. The SQI for patient 23 drops below 20 for the period corresponding to the misled BIS prediction. Despite this, the new index SSE_DoA_ made an appropriate and reasonable estimation for the level of the DoA, which is in alignment with the attending anaesthetist's notes. The divergence in the BIS and the SSE_DoA_ index from 600 to 1300 s is again seen to correspond with a period of poor signal quality. Once the signal quality becomes stable, the new index and the BIS converge at 1400 s.

**Fig. 10:**
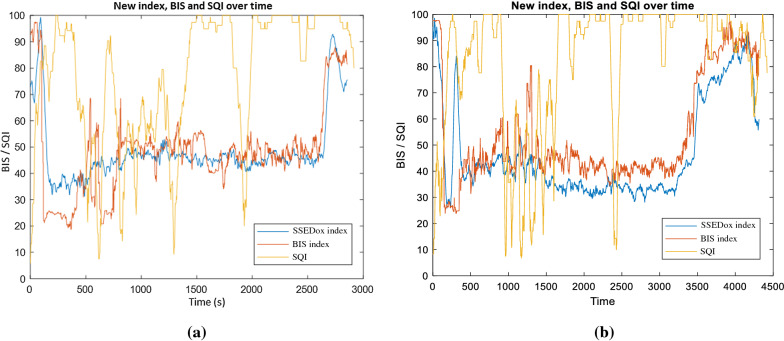
SSE_DoA_ index compared to the BIS with SQI indicated for patient 21 (**a**) and 22 (**b**)

A similar trend is observed in patients 21 and 22, as shown in Fig. 10a and b. The correlation of the SSE_DoA_ index with the BIS value for patient 21 is 0.7916 over the entire surgical procedure. Over this time frame, the mean SQI for patient 36 is seen to be 77.53 with high volatility. Figure 10a illustrates where the volatility in SQI is reflected in the BIS index from 400 to 1500 s. In comparison, the new index is observed to remain relatively stable. Despite the observed poor signal quality for patient 22, BIS and the SSE_DoA_ index correlation is 0.864. The high correlation shown in Fig. 10 suggests its stability in this new index despite signal interference. This is reinforced by the relative stability in the SSE_DoA_ index, compared to the BIS, from 1000 to 1500 s during the period of poor signal quality. Based on the Anaesthetist observations, there is no evidence suggesting that the patient experienced an elevated level of awareness during this period, as indicated by the change in the BIS index.

### Time delay for DoA stage transition

**Fig. 11:**
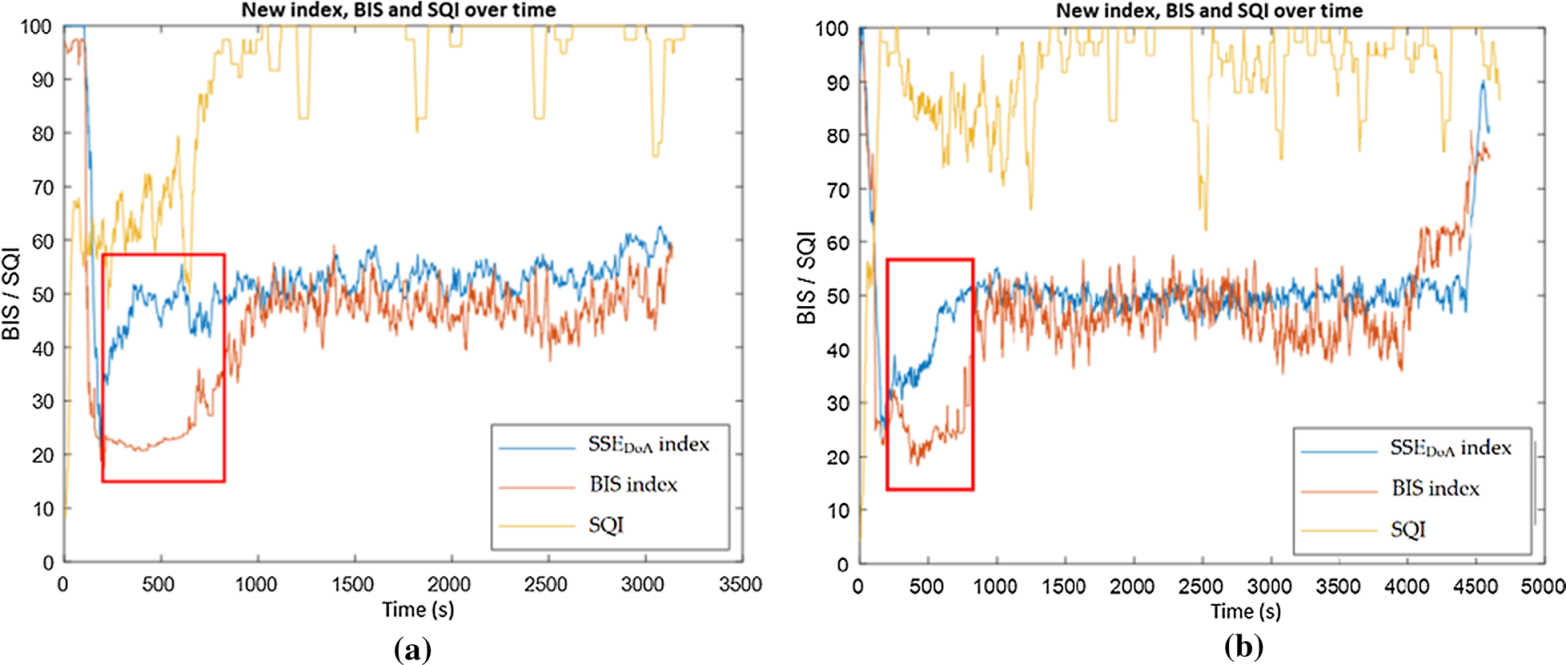
SSE_DoA_ alongside the BIS index with time delay highlighted for **a** for patient 15 and **b** for patient 19

Time delay and responsiveness of a DoA index to changes in a patient's level of awareness are critical for effective surgical awareness management. The newly developed index is seen to exhibit advantages over the BIS index in periods of state changes with extensive time delay observed from the BIS value compared to the. This is evident in patient 15 in Fig. 11a, where the SSE_DoA_ index indicates a change of anaesthetic state from 20 to 50 up to 500 s earlier than the BIS index. This anaesthetic state corresponds to a change from deep to moderate anaesthesia; timely monitoring of these changes is critical during surgical procedures. During this observed time delay, the patient's signal is subject to reduced signal quality. This lower SQI is suggested to be the contributing factor behind the time delay. Due to the significance of this change in SQI, patient 15 was excluded from the main testing patient group.

A similar trend was observed in patient 19 between 400 and 640 s. The time delay of the BIS index compared to the SSE_DoA_ index, in this case, is 239 s, again, during the transition from deep to moderate anaesthesia, as shown in Fig. 11b. In a similar manner to patient 15, this time delay coincided with observed changes in the SQI. These trends have been observed in several other patients, as shown in Table 3.

**Table 3:** Catalogue of time advantage of the SSE_DoA_ index observed across all testing cases

Patient ID	Time delay (s)	Correlation
13	601	0.7861
15	599	0.8124
16	150	0.9427
18	450	0.638
19	239	0.787
20	123	0.8475

In five of the nine test cases, the time delay was observed in the BIS index compared to the SSE_DoA_ index. These effects are outlined in Table 3. In no case was the new SSE_DoA_ index seen to respond to changes in DoA at a slower rate than the BIS index. It is suggested that the time delay seen with the BIS results from changes in signal quality occurring during or just prior to the observed time delay event. The newly developed index SSE_DoA_ is seen to respond to changes in SQI in a superior manner to the BIS index, which exhibits both volatility and time delay during these events.

## Discussion and conclusions

This paper investigated a new DoA index, SSE_DoA,_ based on current and novel practices in EEG signal analysis. Specifically, the inclusion of empirical wavelet transformation is a means of filtering and segmenting the EEG signals. This feature was utilised in combination with SODP and spectral entropy to produce the new DoA index. The outcomes of this research are evaluated against the known DoA industry standard, the BIS. The index was developed utilising the SVM modelling technique and was seen to accurately model changes in the anaesthetic state with an average correlation of 0.834 with the BIS index.

This index was shown to have the capacity to closely follow the BIS index distribution under anaesthesia and, in some cases, exhibited significant time advantages to the BIS. A strong association between the BIS index and the SSE_DoA_ index was observed, with correlations ranging between 0.77 and 0.94 across the testing group. The effect of poor signal quality on the performance of the proposed index was investigated. It was seen that the new index was able to respond to poor signal quality in a superior manner to the BIS index. In these cases, time delay and index volatility were frequently present in the BIS index. These abnormalities were not seen in the SSE_DoA_ index. It is suggested that the effectiveness of the new index, SSE_DoA_, in responding to low levels of signal quality is a direct manifestation of the design of the new index. The adaptability of the EWT method allowed for the proposed support vector machine to deliver robust and meaningful information even when variations in signal quality are present.

This model successfully extracted critical information from the EEG signal to accurately model the state of unconsciousness, as indicated by Cohen's Kappa score of 0.801. This included correspondence with DoA states and transitions and exhibited proficiency during periods of poor signal quality. Furthermore, the new index highlighted time delay in the BIS index corresponding to faster response times during the transition between anaesthetic states. Most significantly, these results were achieved without the application of a discreet denoising process. The advantage of developing a highly effective and adaptable DoA model without a discreet denoising process is significant because of the reduced computational time and processing requirements necessary in developing these surgical applications. Future work in this area will be to explore alternate model building techniques, such as K-Nearest Neighbour (KNN), Support Vector Regression (SVR), or quadratic Gaussian regression.
